# Practical guidelines for B-cell receptor repertoire sequencing analysis

**DOI:** 10.1186/s13073-015-0243-2

**Published:** 2015-11-20

**Authors:** Gur Yaari, Steven H. Kleinstein

**Affiliations:** Bioengineering Program, Faculty of Engineering, Bar-Ilan University, 5290002 Ramat Gan, Israel; Interdepartmental Program in Computational Biology and Bioinformatics, Yale University, New Haven, CT 06511 USA; Departments of Pathology and Immunobiology, Yale University School of Medicine, New Haven, CT 06520 USA

## Abstract

High-throughput sequencing of B-cell immunoglobulin repertoires is increasingly being applied to gain insights into the adaptive immune response in healthy individuals and in those with a wide range of diseases. Recent applications include the study of autoimmunity, infection, allergy, cancer and aging. As sequencing technologies continue to improve, these repertoire sequencing experiments are producing ever larger datasets, with tens- to hundreds-of-millions of sequences. These data require specialized bioinformatics pipelines to be analyzed effectively. Numerous methods and tools have been developed to handle different steps of the analysis, and integrated software suites have recently been made available. However, the field has yet to converge on a standard pipeline for data processing and analysis. Common file formats for data sharing are also lacking. Here we provide a set of practical guidelines for B-cell receptor repertoire sequencing analysis, starting from raw sequencing reads and proceeding through pre-processing, determination of population structure, and analysis of repertoire properties. These include methods for unique molecular identifiers and sequencing error correction, V(D)J assignment and detection of novel alleles, clonal assignment, lineage tree construction, somatic hypermutation modeling, selection analysis, and analysis of stereotyped or convergent responses. The guidelines presented here highlight the major steps involved in the analysis of B-cell repertoire sequencing data, along with recommendations on how to avoid common pitfalls.

## B-cell receptor repertoire sequencing

Rapid improvements in high-throughput sequencing (HTS) technologies are revolutionizing our ability to carry out large-scale genetic profiling studies. Applications of HTS to genomes (DNA sequencing (DNA-seq)), transcriptomes (RNA sequencing (RNA-seq)) and epigenomes (chromatin immunoprecipitation sequencing (ChIP-seq)) are becoming standard components of immune profiling. Each new technique has required the development of specialized computational methods to analyze these complex datasets and produce biologically interpretable results. More recently, HTS has been applied to study the diversity of B cells [1], each of which expresses a practically unique B-cell immunoglobulin receptor (BCR). These BCR repertoire sequencing (Rep-seq) studies have important basic science and clinical relevance [2]. In addition to probing the fundamental processes underlying the immune system in healthy individuals [3–6], Rep-seq has the potential to reveal the mechanisms underlying autoimmune diseases [7–13], allergy [14–16], cancer [17–19] and aging [20–23]. Rep-seq may also shed new light on antibody discovery [24–27]. Although Rep-seq produces important basic science and clinical insights [27], the computational analysis pipelines required to analyze these data have not yet been standardized, and generally remain inaccessible to non-specialists. Thus, it is timely to provide an introduction to the major steps involved in B-cell Rep-seq analysis.

**Fig. 1:**
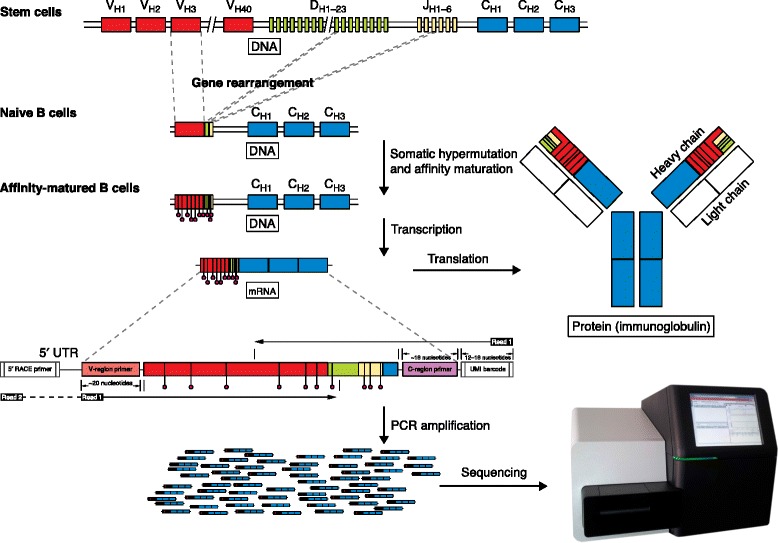
An overview of repertoire sequencing data production. The B-cell immunoglobulin receptor (BCR) is composed of two identical heavy chains (generated by recombination of V, D and J segments), and two identical light chains (generated by recombination of V and J segments). The large number of possible V(D)J segments, combined with additional (junctional) diversity introduced by stochastic nucleotide additions/deletions at the segment junctions (particularly in the heavy chain), lead to a theoretical diversity of >10^14^. Further diversity is introduced into the BCR during adaptive immune responses, when activated B cells undergo a process of somatic hypermutation (SHM). SHM introduces point mutations into the DNA coding for the BCR at a rate of ~10^−3^ per base pair per division [119, 120]. B cells accumulating mutations that improve their ability to bind pathogens are preferentially expanded in a process known as affinity maturation. The biology underlying these processes has been reviewed previously [121]. BCR repertoire sequencing (Rep-seq) experiments can be carried out on mRNA (shown here) or genomic DNA. Sequencer image: A MiSeq from Illumina/Konrad Förstner/Wikimedia Commons/Public Domain. *5′ RACE* 5′ rapid amplification of cDNA ends, *UMI* unique molecular identifier, *5′ UTR* 5′ untranslated region

There are approximately 10^10^–10^11^ B cells in a human adult [28]. These cells are critical components of adaptive immunity, and directly bind to pathogens through BCRs expressed on the cell surface. Each B cell expresses a different BCR that allows it to recognize a particular set of molecular patterns. For example, some B cells will bind to epitopes expressed by influenza A viruses, and others to smallpox viruses. Individual B cells gain this specificity during their development in the bone marrow, where they undergo a somatic rearrangement process that combines multiple germline-encoded gene segments to produce the BCR (Fig. 1). The large number of possible V(D)J segments, combined with additional (junctional) diversity, lead to a theoretical diversity of >10^14^, which is further increased during adaptive immune responses, when activated B cells undergo a process of somatic hypermutation (SHM). Overall, the result is that each B cell expresses a practically unique receptor, whose sequence is the outcome of both germline and somatic diversity.

This review will focus on the analysis of B-cell Rep-seq data sets. Rep-seq studies involve large-scale sequencing of DNA libraries, which are prepared by amplifying the genomic DNA (gDNA) or mRNA coding for the BCR using PCR (Fig. 1). The development of HTS technologies and library preparation methods for Rep-seq is an area of active research, and has been reviewed elsewhere [1, 29]. While the experimental technologies and analysis methods are in a phase of rapid evolution, recent studies share common analysis tasks. Many of these steps also apply to the analysis of T-cell receptor sequencing data, and these should be standardized and automated in the future. The development of software toolkits, such as pRESTO/Change-O [30, 31], take a step in this direction by providing independent modules that can be easily integrated. For bioinformaticians and others used to dealing with different types of HTS experimental data (such as DNA-seq and RNA-seq data), approaching Rep-seq data requires a change of mindset. First, BCR sequences are not encoded directly in the genome. While parts of the BCR can be traced back to segments encoded in the germline (that is, the V, D and J segments), the set of segments used by each receptor is something that needs to be inferred, as it is coded in a highly repetitive region of the genome and currently cannot be sequenced directly. Furthermore, these segments can be significantly modified during the rearrangement process and through SHM, which leads to >5 % of bases being mutated in many B-cell subsets. Thus, there are no pre-existing full-length templates to align the sequencing reads.

**Fig. 2:**
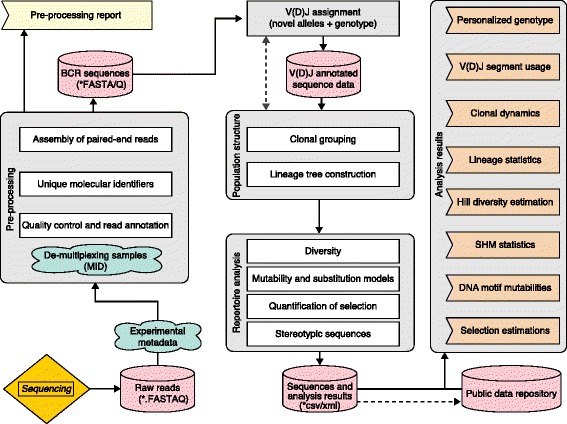
The essential steps in repertoire sequencing analysis. Repertoire sequencing (Rep-seq) analysis can be divided into three stages: pre-processing; inference of B-cell population structure; and detailed repertoire analysis. Pre-processing transforms the next-generation sequencing reads into error-corrected B-cell immunoglobulin receptor (*BCR*) sequences, which are then aligned to identify the V(D)J germline genes. Next, the dynamic population structure of the BCR repertoire is inferred. Finally, quantitative features of the B-cell repertoire are calculated. *MID* multiplex identifier, *SHM* somatic hypermutation

This review aims to provide step-by-step guidance to fundamental aspects of B-cell Rep-seq analysis. The analysis is divided into three stages: pre-processing of sequencing data, inference of B-cell population structure, and detailed repertoire analysis (Fig. 2).

## Pre-processing

The goal of the pre-processing stage is to transform the raw reads that are produced by HTS into error-corrected BCR sequences. As discussed below, factors such as sequencing depth, read length, paired-end versus single-end reads, and inclusion of unique molecular identifiers (UMIs; sometimes referred to as UIDs) affect the analysis steps that need to be taken. Pipelines will need to be run many times to determine the proper parameters and data flow. Therefore, if the data are very large (several million reads per sample are common), it is advisable to sample a random subset (say 10,000 reads) and carry out the steps below to make sure quality is reasonable and the read conforms to the experimental design. Once the analysis steps are integrated, and the parameters are fixed, the pre-processing pipeline can be run on the full data set. It is useful to keep track of how many sequences pass each step successfully so that outliers can be detected. The outliers may reflect steps for which the parameters need further tuning or may indicate issues related to the experiments. We split the pre-processing stage into three steps: quality control and read annotation; UMIs; and assembly of paired-end reads.

### Quality control and read annotation

The typical starting point for pre-processing is a set of FASTQ (or FASTA) files [32], and the tools used in this stage of the analysis often utilize this file format. Throughout processing, sequence-level annotations will be accumulated (for example, average quality, primers used, UMIs, and so on). These annotations can be stored in a database and linked to the reads within the FASTQ files through a lookup table. An alternative is to propagate the accumulated annotations within the read headers, thus maintaining all the data together in the FASTQ format [30]. If samples are multiplexed, the sequencing facility will normally de-multiplex the data into one FASTQ file for each sample. If the data are paired-end, each sample will produce two FASTQ files (one for each read-end). If the data have not been de-multiplexed by the sequencing facility, the first step in the analysis is to identify the sample identification tags (often referred to as multiplex identifiers (MIDs) or sample identifiers (SIDs)) to determine which reads belong to which samples. These MID tags typically consist of a short number of base pairs (commonly 6–16) that are located near the end(s) of the amplicon. If multiple MIDs are designed to be in each sequence, these should be checked for consistency in order to reduce the probability of misclassification of reads due to PCR and sequencing errors [33].

Individual reads differ in quality, which is measured at the base level using Phred-like scores [34]. Read quality metrics can be computed and visualized with software such as FastQC [35]. It is important to remember that the quality estimates output by the sequencer do not account for errors introduced at the reverse transcription and PCR amplification steps. It is desirable to have a Phred-like score >30 for a long stretch at the beginning of each read. Quality will typically drop near the end of each read [36]. If the library is designed to have a lot of overlap in the paired reads, then low-quality positions at the ends of the reads can be cut at this stage to allow better assembly of the paired reads. Some reads will have overall low quality, and sequences with low average quality (for example, less than a threshold of ~20) should be removed. A Phred-like score of 20 means 1 error per 100 base pairs (*p* = 10^−*Q*/10^), where *p* is the probability of an erroneous base call and *Q* is the Phred-like score associated with this base). The appropriate quality thresholds to employ are dataset dependent, and insight may be gained by plotting the distribution of quality scores as a function of position in the sequence. Although more stringent quality cutoffs will lower the number of sequences, it is crucial to keep quality high in Rep-seq data since BCR sequences can differ from one another by single nucleotides.

After handling low-quality reads and bases, reads can be analyzed to identify, annotate, and mask the primers used. The location of the primer sequences depends on the library preparation protocol. A typical setup includes a collection of V segment primers at the 5′ end and a set of J (or constant region) primers at the 3′ end of the amplicon (Fig. 2). In library preparation protocols in which 5′ rapid amplification of cDNA ends (5′ RACE) is used, there will not be a V segment primer [37, 38]. Primers are identified by scoring the alignment of each potential primer to the read and choosing the best match. In this step, it is crucial to know where on the read (and on which read of a pair) each primer is located. Even when primers are expected to be at a particular location in the read, they may be off by a few bases due to insertions and deletions (indels). If searching for primers within a range of locations, plotting a histogram of the identified locations is recommended to make sure this conforms to experimental design. Reads produced by sequencing may be in unknown orientations, depending on the experimental protocol. In this case, primers may appear in a forward or reverse orientation (and on either read for a paired-end setup). In cases where the primer is found in the reverse complement orientation, it is a good idea to reverse complement the sequence so that all reads are in the same orientation for the remaining analysis steps.

Primers are typically associated with some information, which should be used to annotate the reads. For example, each constant region primer may be associated with a specific isotype (immunoglobulin (Ig)M, IgG, and so on). The part of the sequence that matches the primer should then be cut or masked (bases changed to N). This is because the region bound by the primer may not accurately reflect the state of the mRNA/DNA molecule being amplified. For example, a primer designed to match a germline V segment sequence may bind to sequences with somatic mutations, thus leading to inaccuracy in mutation identification in downstream analysis. Reads for which primers cannot be identified (or do not appear in the expected locations) should be discarded. When dealing with paired-end data, annotations need to be kept in sync between the read pairs. If discarding one read of a pair, it may be necessary to also discard the other read of the pair (if later steps of the analysis depend on having both ends). Several tools for this step include PANDAseq [39], PEAR [40], pRESTO [30], and USEARCH [41] (for a broader list and comparison of features see [30]).

### Unique molecular identifiers

UMIs are highly diverse nucleotide tags appended to the mRNA, usually at the reverse transcription step [42]. UMIs are usually located at a specific position(s) in a read (for example, a 12 base pair (bp) UMI at one end of the read or split as two 6 bp identifiers at opposite ends of the amplicon). The length of the UMI depends on protocol, but is typically around 15 bases [12, 42, 43]. The random nature of the UMI enables each sequence to be associated with a single mRNA molecule. They are designed to reduce PCR amplification biases and sequencing error rates through the generation of consensus sequences from all amplicons with the same UMI.

UMI information is first identified in each read, and then it is removed from the read and the read is annotated with the UMI sequence. Next, it should be checked that the UMIs conform to the experimental protocol by plotting the distribution of bases at each position in the UMI and the distribution of reads per UMI to make sure that there are no unexpected biases. It is possible for an mRNA molecule to end up with multiple UMIs owing to the accumulation of PCR and sequencing errors in the UMI. Important factors here include UMI length (the longer it is, the higher the potential for errors, while shorter UMIs reduce diversity), and the number of PCR cycles (more cycles increase the potential for errors). Thus, sequences with “similar” UMIs should be clustered together. To get a sense of the extent to which UMI errors affect the analysis for particular data sets, “distance-to-nearest” plots [18] can be made for the UMI. If two peaks are observed, the first peak is interpreted as the distance between UMIs originating from the same molecule, while the second peak reflects the distance between UMIs that originated from distinct molecules. Clustering approaches can be used for recognizing UMIs that are expected to correspond to the same pre-amplified mRNA molecule (for example, single linkage hierarchical clustering). However, it is possible that each of these UMI clusters corresponds to multiple mRNA molecules. This may be due to incorrect merging, insufficient UMI diversity (that is, UMI sequences that are too short, or bad quality such as GC content biases), or bad luck [44]. Thus, when merging multiple UMIs into a single cluster, checking that the rest of the sequence is also similar is recommended. The sequences within the cluster would be expected to differ only due to PCR and sequencing errors. A second clustering step should be carried out on UMI clusters with high diversity, to further partition the sequences based on the non-UMI part of the reads.

Once the reads are partitioned into clusters, each corresponding to a single mRNA molecule, the next step is to build a consensus sequence from each cluster of reads. The consensus sequence utilizes information from all reads in the cluster and thus improves the reliability of the base calls. This can take into account the per-base quality scores, which can be propagated to the consensus sequence. Maintaining the quality scores and the number of reads can help in filtering steps later in the analysis. Overall, each UMI cluster results in a single consensus sequence (or two in paired-end setups). Available tools for this step include MiGEC [45] and pRESTO [30].

### Assembly of paired-end reads

The length of the PCR amplicons being sequenced in a Rep-seq experiment varies considerably because the BCR sequences use different V, D and/or J segments, which can vary in length. Nucleotide addition and deletion at the junction regions further alters the sequence length distribution. For examples of length distributions see [46]. Also, sequence lengths depend on where the primers are located, and can differ for each primer (for example, isotype primers may be at different locations relative to the V(D)J sequence). In most cases, experiments using paired-end sequencing are designed so that the two reads are expected to overlap each other. The actual extent of overlap depends on the BCR sequence and read length. Assembly of the two reads into a single BCR sequence can be done de novo by scoring different possible overlaps and choosing the most significant. Discarding reads that fail to assemble may bias the data towards shorter BCR sequences, which will have a longer overlapping region. When the overlap region is expected to be in the V segment, it is also possible to determine the relative positions of the reads by aligning them to the same germline V segment. This is especially useful when not all read pairs are expected to overlap, and Ns can be added between the reads to indicate positions that have not been sequenced. Several tools can be used to assemble paired-end reads [30, 39, 40]. As quality control, it is a good idea to analyze the distribution of overlap lengths to identify outliers. Since each read of a pair may be associated with different annotations (for example, which primers were identified), it is critical to merge these annotations so that they are all associated with the single assembled read. Similar to the case described earlier in which reads with the same UMI were merged, the base quality in the overlap region can be recomputed and propagated. At this point, another quality filtering step can be undertaken. This could include removing sequences with a low average quality, removing sequences with too many low-quality individual bases, or masking low-quality positions with Ns. For efficiency of the next steps, it is also useful to identify sequences that are identical at the nucleotide level, referred to as “duplicate” sequences, and group them to create a set of “unique” sequences. Identifying duplicate sequences is non-trivial when degenerate nucleotide symbols are present, since there may be multiple possible groupings (consider AN, AT and NT) or the consensus may create a sequence that does not exist (consider AN and NT). When grouping duplicate sequences, it is important to propagate annotations, and keep track of how much support there is for each unique sequence in the underlying data. To improve quality, each unique mRNA should be supported by a minimum level of evidence. One approach is to require a minimum number for the raw reads that were used to construct the sequence (for example, two). A more stringent approach could also require a minimum number of independent mRNA molecules (for example, two UMIs). This could help to control for errors at the reverse transcription step [45], at the expense of sequences with low BCR expression.

## V(D)J germline segment assignment

In order to identify somatic mutations, it is necessary to infer the germline (pre-mutation) state for each observed sequence. This involves identifying the V(D)J segments that were rearranged to generate the BCR and determining the boundaries between each segment. Most commonly this is done by applying an algorithm to choose among a set of potential germline segments from a database of known segment alleles. Since the observed BCR sequences may be mutated, the identification is valid only in a statistical sense. As such, multiple potential germline segment combinations may be equally likely. In these cases, many tools for V(D)J assignment report multiple possible segments for each BCR sequence. In practice, it is common to use one of the matching segments and ignore the rest. This has the potential to introduce artificial mutations at positions where the possible segments differ from each other. Genotyping and clonal grouping, which are described below, can help reduce the number of sequences that have multiple segment assignments. For sequences that continue to have multiple possible germline segments, the positions that differ among these germline segments should be ignored when identifying somatic mutations, for example, by masking the differing position(s) in the germline with Ns.

There have been many approaches developed for V(D)J assignment [47–52]. Important features that distinguish these tools include web-based versus stand-alone versions, allowing the use of an arbitrary germline segment database, computing time, the quality of D segment calls, allowing multiple D segments in a single rearrangement, allowing inverted or no D segments, and the availability of source code. This is an active field of research, with each tool having particular strengths and weaknesses depending on the evaluation criteria and assumptions about the underlying data. Methods continue to be developed, and contests have even been run to inspire the development of improved methods [53]. In general, V and J assignments are much more reliable than D segment assignments, as the D regions in BCR sequences are typically much shorter and highly altered during the rearrangement process.

The performance of V(D)J assignment methods crucially depends on the set of germline V(D)J segments. If the segment allele used by a BCR does not appear in the database, then the polymorphic position(s) will be identified as somatic mutation(s). The most widely used database is IMGT [47], and requires significant evidence to include alleles, while other databases such as UNSWIg have been developed to include alleles with less stringent criteria [54]. However, it is clear from recent studies that the number of alleles in the human population is much larger than the number covered by any of these databases [55–57]. Identification of germline segments for other species is an active area of study [58–61], and these too are likely to expand over time. Thus, an important step in the analysis is to try and identify novel alleles directly from the data being analyzed using tools such as TIgGER [57]. Determining haplotypes [62] can further improve V(D)J assignment by restricting the allowed V–J pairings. Determining the genotype of an individual can significantly improve the V(D)J assignment quality. Genotypes can be inferred either by studying sequences with low mutation frequencies or from sorted naive cells [5, 57]. In the future, it may be possible to obtain the set of germline alleles for an individual directly from DNA sequencing of non-B cells. Currently this is not possible as the region of the genome encoding these segments is highly repetitive and aligning short reads to it is challenging. However, as read lengths increase and alignment algorithms are further developed this is expected to be feasible in the near or intermediate future.

Once the V(D)J germline segments have been assigned, indels in the BCR sequence can be identified within these segments. Several methods assume that any identified indels in the V/J segments are the result of sequencing error, and will “correct” them (for example, by introducing a gap for deletions or removing insertions). Indels can occur during affinity maturation [63], although the frequency of occurrence is not yet clear, and these can be lost with many computational pipelines.

Having determined the germline state, it is common to partition the sequences into functional and non-functional groups. Non-functional sequences are defined by characteristics including: having a frameshift between the V and J segments; containing a stop codon; or containing a mutation in one of the invariant positions. These non-functional sequences may represent real sequences that were non-productively rearranged or acquired the modification in the course of affinity maturation. However, many are likely the result of experimental errors, especially when the data are derived from sequencing platforms that are prone to introducing indels at high rates in photopolymer tracts. It is common to discard non-functional sequences from the analysis. If it is desired to analyze non-productively rearranged sequences, it is important to focus on the subset of non-functional sequences that are most likely to have been produced during the rearrangement process (for example, those having frameshifts in the junction areas separating the V–D and D–J segments identified as N-additions or P-additions [64]).

## Population structure

Clonal expansion and affinity maturation characterize the adaptive B-cell response. The goal of this stage is to infer the dynamic population structure that results from these processes. Available tools for inferring population structure include Change-O [31], IgTree [65], and MiXCR [66]. In this section we split the population structure inference stage into two steps: clonal grouping and B-cell lineage trees.

### Clonal grouping

Clonal grouping (sometimes referred to as clonotyping) involves clustering the set of BCR sequences into B-cell clones, which are defined as a group of cells that are descended from a common ancestor. Unlike the case for T cells, members of a B-cell clone do not carry identical V(D)J sequences, but differ because of SHM. Thus, defining clones based on BCR sequence data is a difficult problem [67, 68]. Methods from machine learning and statistics have been adapted to this problem. Clonal grouping is generally restricted to heavy chain sequences, as the diversity of light chains is not sufficient to distinguish clones with reasonable certainty. As newer experimental protocols allow the determination of paired heavy and light chains [69, 70], these can both be combined.

The most basic method for identifying clonal groups involves two steps. First, sequences that have the same V and J segment calls, and junctions of the same length, are grouped. Second, the sequences within each group are clustered according to a sequence-based distance measure. Most commonly, the distance measure is focused on the junction region, and is defined by nucleotide similarity. When calculating this “hamming distance”, it is important to account for degenerate symbols (for example, Ns). Although it is common to look for clonal variants only among sequences that have junction regions of the same length, it is possible that SHM can introduce indels during the affinity maturation process [63]. Clonal groups should be defined using nucleotide sequences, and not amino acids, since the rearrangement process and SHM operate at the nucleotide level. Moreover, convergent evolution can produce independent clonal variants with similar amino acid sequences [71, 72]. Other distance measures have been proposed that take into account the intrinsic biases of SHM [31]. The idea behind these methods is that sequences that differ at an SHM hotspot position are more similar than those that are separated by a coldspot mutation. Given a distance measure, clustering can be done with standard approaches, such as hierarchical clustering using single, average or complete linkage. Each of these methods require a distance cutoff. This is commonly determined through inspection of a “distance-to-nearest” plot [18]. An alternative to the clustering approach is to construct a lineage tree (see below), and cut the tree to create sub-trees, each of which corresponds to a clonal group [73]. Maximum likelihood approaches have also been used [63, 74]. So far, there have not been rigorous comparisons of these methods. Once the clonal groups have been determined, these can be used to improve the initial V(D)J allele assignments, as all the sequences in a clone arise from the same germline state [75]. In principle, clustering sequences into clones can also be done before or in parallel with V(D)J assignments [76].

It is important to consider the set of sequences on which clonal grouping is carried out. For example, if cells are collected from multiple tissues or different sorted B-cell subsets, these can be merged together before analysis to identify clonal groups that span multiple compartments. Sometimes reference sequences are also available (for example, antigen-specific sequences from other samples of the same subject [15, 77] or from the literature [72]), and these may also be added to the set of sequences. As the clonal groups can change depending on the full set of data, it is important to be consistent in the choice of data being used for the analysis. Clonal grouping could also be impacted by experimental factors such as sampling and sequencing depth. Two members of a clone that differ significantly may only be recognized as such if intermediate members — that share mutations with both — are sequenced. By definition, clones cannot span different individuals. Thus, looking at the frequency of clones that are shared across individuals can provide a measure of specificity for the clonal grouping method. Although so-called “public” junction sequences have been observed, these tend to be rare (at least in heavy chains) [18].

### B-cell lineage trees

B-cell lineage trees are constructed from the set of sequences comprising each clone to infer the ancestral relationships among individual cells. The most frequently applied methods are maximum parsimony and maximum likelihood, which were originally developed in evolutionary biology [78]. Briefly, maximum parsimony attempts to minimize the number of independent mutation events, while maximum likelihood attempts to build the most likely tree given a specific nucleotide substitution matrix. These methods were developed using several assumptions, such as long timescales and independent evolution of each nucleotide, which do not hold for B-cell affinity maturation. Significant work remains to be done in order to validate and adapt these methods to B-cell Rep-seq analysis. Nevertheless, the existing approaches still form the basis for current Rep-seq studies. Many tools exist in evolutionary biology for phylogenetic tree construction [79–81]. The output of these tools is usually modified in B-cell trees to reflect common conventions in immunology, such as allowing observed sequences to appear as internal nodes in the tree and listing the specific nucleotide exchanges associated with each edge. Insights can be obtained by overlaying other sequence-specific information on the tree, including mutation frequencies [82], selection strengths [83], number of mRNAs observed [12], isotype [13, 14], or tissue location [9, 12, 77]. Lineage trees provide information on the temporal ordering of mutations, and this information can be used along with selection analysis methods to study temporal aspects of affinity maturation [73, 84, 85]. Quantitative analysis of lineage tree topologies has also been used to gain insights into the underlying population dynamics [86] and cell trafficking patterns between tissues [12, 13, 87]. In most current pipelines, grouping the sequences into clones and constructing lineage trees are separate steps. However, they are highly related and future methods may integrate these two steps.

## Repertoire analysis

**Fig. 3:**
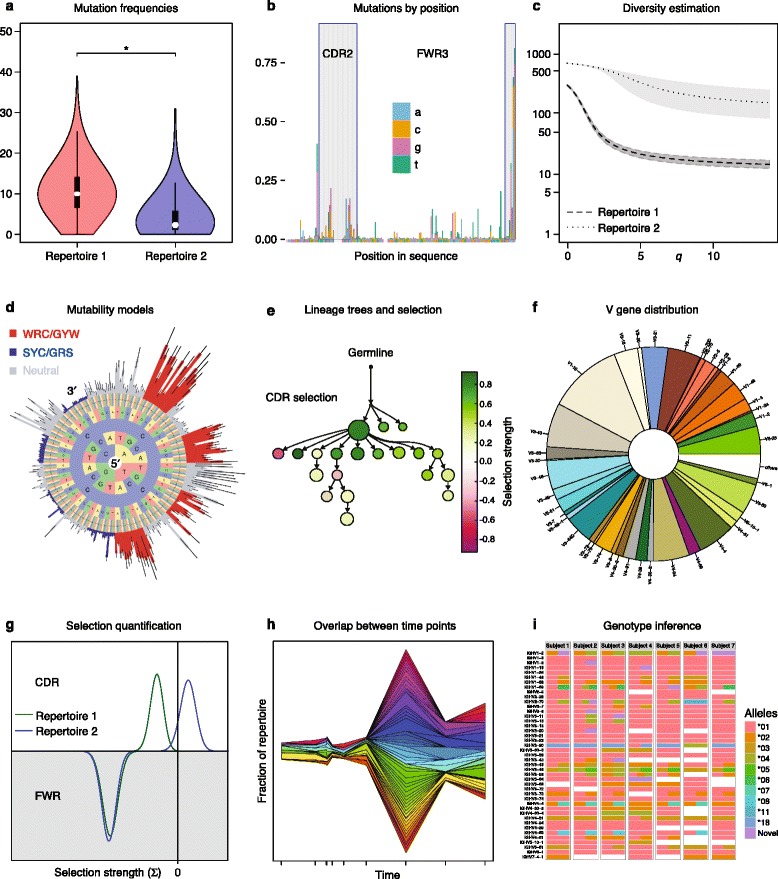
Example outcomes of repertoire sequencing analysis. **a** A violin plot comparing the distribution of somatic mutation frequencies (across B-cell immunoglobulin receptor (BCR) sequences) between two repertoires. **b** The observed mutation frequency at each position in the BCR sequence, with the complementarity determining regions (*CDRs*) indicated by *shaded areas*. **c** Comparing the diversity of two repertoires by plotting Hill curves using Change-O [31]. **d** A “hedgehog” plot of estimated mutabilities for DNA motifs centered on the base cytosine (C), with coloring used to indicate traditional hot- and coldspots. **e** A lineage tree with superimposed selection strength estimates calculated using BASELINe [110]. **f** Pie chart depicting V segment usage for a single repertoire. **g** Comparison of selection strengths in two repertoires by plotting the full probability density function for the estimate of selection strength (calculated using BASELINe) for the CDR (*top*) and framework region (*FWR*; *bottom*). **h** Stream plot showing how clones expand and contract over time. **i** V segment genotype table for seven individuals determined using TIgGER [57]

The goal of this stage is to calculate quantitative features of the B-cell repertoire that can further be utilized for different aims such as: classification of data from different cohorts; isolating specific BCR populations for further study (for example, drug candidates); and identifying active and conserved residues of these specific BCR sequences. Effective visualizations are crucial to simplify these high-dimensional data, and Rep-seq analysis methods are associated with different types of plots that highlight specific features of these data (Fig. 3).

### Diversity

Estimating repertoire diversity, and linking changes in diversity with clinical status and outcomes is an active area of research [88, 89]. Multiple diversity measures have been studied intensively in the field of ecology, and many of the attempts that have been made so far to characterize diversity in immune repertoires have used these concepts and methods. In ecological terms, an individual animal is the analogue of a B cell while a species is the analogue of a clone. All diversity analyses begin from a table of clonal group sizes. Traditionally, the three main diversity measures are species richness, the Shannon entropy, and the Gini–Simpson index. Each reflects different aspects of diversity and has biases when applied to particular underlying populations in terms of size and abundance distribution. When two populations (repertoires in our case) are being compared, it can be the case that one diversity measure shows a certain trend while the other shows the opposite since they represent different aspects of the underlying abundance distributions [89]. Moreover, these measures are dependent on the number of sampled B cells. Thus, sampling issues need to be addressed before diversity measures are compared. One strategy is to subsample the larger repertoire to the size of the smaller one and compare the two [12]. Another approach is to interpolate the diversity measure for smaller sampling sizes and then to extrapolate from these subsamples the asymptotic values of each of the samples and compare them [90]. It is important to note that when a repertoire is subsampled, the partitioning of sequences into clones needs to be redone on each subsampled population as clone definitions are influenced by sampling depth. In order to capture more information about the full clone size distribution, use of the Hill family of diversity indices has been advocated [91, 92]. The Hill indices are a generalization of the three measures mentioned above, and define diversity as a function of a continuous parameter *q. q* = 0 corresponds to clonal richness (number of clones), *q* = 1 is the exponential of the Shannon index, *q* = 2 is the reciprocal of the original Simpson index or one minus the Gini–Simpson index, and as *q* approaches infinity, the corresponding Hill index approaches the reciprocal of the largest clone frequency. Subsampling approaches can also be applied to the full Hill curve [90], resulting in a powerful set of repertoire features that can be used to characterize cells from different subsets, tissues, or disease states [89].

In the above discussion, clonal abundances were defined by the number of B cells in each clone. However, this is usually not measured directly. The mRNAs being sequenced are commonly pooled from many individual cells. Thus, observing multiple occurrences of the same sequence could be caused by PCR amplification of a single mRNA molecule, sampling multiple molecules from the same cell, or multiple cells expressing the same receptor. One strategy to estimate diversity is to group identical sequences together and analyze the set of unique sequences (these groups can be defined to include sequences that are similar as well to account for possible sequencing errors [33]). If each unique sequence corresponds to at least one independent cell, this provides a lower bound on diversity and other repertoire properties. Including UMIs in the experimental method helps to improve the diversity estimation by correcting for PCR amplification. However, some bias may be introduced because different cell subsets can express widely varying levels of BCR gene mRNAs, with antibody-secreting cells being especially high [93]. Sequencing from multiple aliquots of the same sample can be used to estimate the frequency of cells expressing the same receptor [94]. Emerging single-cell technologies will eventually provide a direct link between sequences and cells [70, 95], and may also provide insight into the contribution of transcription errors, estimated to be ~10^−4^ [96], to the observed mRNA diversity.

### Somatic hypermutation

During adaptive immune responses, B cells undergo a process of SHM. Thus, even cells that are part of the same clone can express different receptors, which differs from T cells, in which all clonal members share the same receptor sequence. A crucial step in B-cell Rep-seq analysis is therefore to identify these somatic mutations. Having identified the germline state of the sequence using the methods described above, somatic mutations are called when the observed sequence and the inferred germline state differ. In carrying out this comparison, it is important to properly account for degenerate nucleotide symbols (that is, a “mismatch” with an N should not be counted as a mutation). It is common to calculate mutation frequencies for the V segment (up to the start of the junction) since the inferred germline state of the junction is less reliable. Mutations in the J segment (after the end of the junction) may also be included in the analysis. Somatic mutation frequencies are expressed in per bp units, so it is important to calculate the number of bases included in the analysis, and not use a per sequence average, in which the number of bases in each sequence may differ (for example, due to different primers, different V segment lengths, or the number of low-quality bases that were masked).

SHM does not target all positions in the BCR equally. There is a preference to mutate particular DNA motifs (hotspots) and not others (coldspots). WRCY is a classic hotspot motif, while SYC is a well-known coldspot motif [97]. However, there is a wide range of mutabilities that depends on the local nucleotide context of each position [98, 99]. Mutability models can be estimated directly from Rep-seq data [99, 100], using tools such as Change-O [31]. These models have a number of uses as differences in mutation patterns may be linked to the various enzymes involved in SHM [101]. Mutability models also provide critical background models for the statistical analysis of selection, as described below. Methods to estimate mutability need to account for biases in the observed mutation patterns due to positive and/or negative selection pressures. Strategies include focusing on the set of non-functional sequences, using intronic sequences, or basing models on the set of silent (synonymous) mutations [99, 102, 103].

The frequency of somatic mutations is not uniform across the BCR. The V(D)J region of the BCR can be partitioned into framework regions (FWRs) and complementarity determining regions (CDRs) [104]. FWRs typically have a lower observed mutation frequency, in part because they code for regions important to maintain structural integrity, and many mutations that alter the amino acid sequence are negatively selected [105]. CDRs have higher observed mutation frequencies, in part because they contain more hotspot motifs and their structure is less constrained. Mutability models can be used to estimate the expected frequency of mutations in different regions of the V(D)J sequence. Deviations from the expectation provide useful biological information. It is common to look for an increased frequency of replacement (non-synonymous) mutations as evidence of antigen-driven positive selection, and a decreased frequency of replacement mutations as evidence of negative selection [106]. Selection analysis has many applications, including the identification of potentially high-affinity sequences, understanding how different genetic manipulations impact affinity maturation, and investigating whether disease processes are antigen driven. Methods to detect selection based on the analysis of clonal lineage trees have also been proposed [107], as well as hybrid methods [108]. Enrichment for mutations at specific positions can also be done by comparing the observed frequency with an empirical background distribution from a set of control sequences [72, 100, 109]. When comparing selection across biological conditions, it is important to remember that lower *P* values do not necessarily imply stronger selection, and methods such as BASELINe [110], which quantifies the strength of selection (rather than simply detecting its presence), should be employed. BASELINe defines selection strength as the log-odds ratio between the expected and observed frequencies of non-synonymous mutations, and estimates a full probability density for the strength using a Bayesian statistical framework. When discussing “selection”, it is important to distinguish between different types of selection that can occur during different phases of B-cell maturation. SHM and affinity maturation are processes that operate on mature B cells during adaptive immune responses. During development, immature B cells progress through several stages and are subject to central and peripheral checkpoints that select against autoreactive cells, leading to biased receptor properties (for example, changes in V segment usage, or the average length of the CDR3 region) [46]. Probabilistic frameworks have been developed to model these properties, allowing them to be compared at various stages of development to determine which properties are influenced by this selection [100].

### Stereotypic sequences and convergent evolution

B cells responding to common antigens may express BCRs with shared characteristics. These are referred to as stereotyped BCRs, and their identification is of significant interest [111]. Stereotypic receptors can reflect germline characteristics (for example, the use of common V, D or J segments), or arise through convergent evolution, in which the accumulation of somatic mutations results in common amino acid sequences. These common patterns may serve as diagnostic markers [112]. Stereotyped receptors have been observed in infections, autoimmunity and cancer [111].

Stereotyped sequences are commonly defined by having similar junctions. One way to observe them is to pool the data from several individuals together before carrying out the clonal grouping step. In this case, the distance function used for clonal grouping can be based on the amino acid sequence, rather than the nucleotide sequence (but note that these results no longer represent true clones). Sets of sequences that span multiple individuals can then be identified and extracted for more focused study. Although they exist, the percentage of such sequences is usually low. Significant overlap across individuals is most often the result of experimental problems, such as sample contamination or MID errors in multiplexed sequencing runs. Identification of shared amino acid motifs across the entire BCR sequence can be carried out using widely used motif finding tools [113]. In these analyses, the choice of a control sequence set is critical and should account for germline segment usage and SHM. When looking for sequences with common features across individuals (or time points), it is important to consider statistical power. If the relevant sequences constitute a small percentage of the repertoire, then the ability to detect such sequences will depend on many experimental factors, including the number and type of cells sampled, the sequencing depth, and cohort heterogeneity. Statistical frameworks for power analysis in Rep-seq studies are lacking, and are an important area for future work.

## Conclusions

Like the experimental technologies used to generate HTS data, the development of Rep-seq analysis methods is a fast-moving field. While computational methods have been developed to address important questions, many of the proposed tools have yet to be rigorously evaluated. Comparative studies, conducted on reference experimental and simulated data, are critical to have a quantitative basis for selecting the best methods to use in each step of the analysis. This will be facilitated by making the source code available for Rep-seq analysis tools, and not only providing web-based interfaces or services. Ideally, the source code should be posted in a public version control repository (such as bitbucket, github, Google source, or others) where bugs and comments can be reported. The community will also be aided by an active platform for informal discussions and evaluation of existing and new tools for Rep-seq analysis. The OMICtools directory [114] provides a promising step in this direction, and includes a dedicated Rep-seq section where a large list of current software tools can be found.

A challenge in developing computational pipelines using the kinds of methods described here is that each tool may require its own input format. Considerable effort is necessary to reformat data. For example, different V(D)J assignment tools can output the “junction sequence” but use different region definitions or numbering schemes. Ontologies can provide a formal framework for standardization of data elements, and a source of controlled vocabularies [115]. A common data format for sequences and results can facilitate data sharing, as well as integration of methods and tools from multiple research groups. Many tools use tab-delimited files for data and analysis results, and XML-based schemes have also been proposed [116]. Standardizing the terms used in column headers, or the XML tags, would greatly enhance interoperability. Some integrated frameworks are emerging, such as pRESTO/Change-O [30, 31], to provide standardized analysis methods in modular formats so that analysis pipelines can be rapidly developed and easily customized.

Many of the steps in Rep-seq analysis are computationally intensive, making them difficult to carry out on standard desktop computers. High-performance computing clusters, cloud-based services, as well as graphics processing unit (GPU)-enabled methods can help relieve this bottleneck. These approaches require programming expertise, or specifically designed tools. Some tools, such as IMGT/HighV-QUEST [47] or VDJServer [117], offer web-based front ends for some analysis steps, in which users can submit data to be analyzed on dedicated servers. For human studies, ethical issues with regards to patient confidentiality (for example, US Health Insurance Portability and Accountability Act (HIPAA) privacy restrictions) and governance over the use of sample-derived data need to be considered before uploading data onto public servers. These considerations are also important when the data are submitted to public repositories. Many current Rep-seq studies are made available through SRA or dbGAP [118], and only the latter has access control.

Novel computational methods continue to be developed to address each new improvement in sequencing technologies. Emerging techniques for high-throughput single-cell analysis (allowing for heavy and light chain pairing) will soon be adapted to sequence multiple genes along with the BCR, and eventually the full genome. This technological progress offers new opportunities for biological and clinical insights, and the computational methods discussed here will continue to evolve in this ongoing effort.

## Abbreviations

5′ RACE: 5′ rapid amplification of cDNA ends
BCR: B-cell immunoglobulin receptor
bp: base pair
cDNA: complementary DNA
CDR: complementarity determining region
ChIP-seq: chromatin immunoprecipitation followed by sequencing
DNA-seq: DNA sequencing
FWR: framework region
gDNA: genomic DNA
GPU: graphics processing unit
HIPAA: Health Insurance Portability and Accountability Act
HTS: high-throughput sequencing
Ig: immunoglobulin
indel: insertion and deletion
MID: multiplex identifier
Rep-seq: repertoire sequencing
RNA-seq: RNA sequencing
SHM: somatic hypermutation
SID: sample identifier
UMI: unique molecular identifier
UTR: untranslated region
